# Etonogestrel implant failure in a woman taking thyroid hormone replacement: A case report

**DOI:** 10.1016/j.crwh.2025.e00687

**Published:** 2025-01-17

**Authors:** Alice Moylan, Jewel Brown, Melissa J. Chen, Mitchell D. Creinin

**Affiliations:** a University of California, Davis School of Medicine, 4610 X St., Sacramento, CA 95817, USA; b Department of Obstetrics and Gynecology, University of California, Davis Health, 4860 Y Street, Suite 2500, Sacramento, CA 95817, USA

**Keywords:** Contraceptive implant, Etonogestrel, Hyperthyroidism, Pregnancy

## Abstract

The etonogestrel implant is known to have high contraceptive efficacy for up to 5 years. This case report describes etonogestrel implant failure during year 4 of use in a patient with a normal body mass index. The patient was receiving thyroid hormone replacement after a thyroidectomy and was found to have iatrogenic thyrotoxicosis in the months preceding pregnancy. Further study of the effects of thyroid hormone on etonogestrel metabolism are indicated.

## Highlights

• Case report of etonogestrel implant failure in year 4 of use in a patient on taking levothyroxine.
• Exogenous thyroid hormone replacement resulted in thyrotoxicosis.
• Thyrotoxicosis may decrease the efficacy of the contraceptive implant.

## 1 Introduction

The etonogestrel implant is one of the most effective forms of contraception, with a pregnancy rate of approximately 0.4 per 100 women-years during the first 3 years of use [1]. The implant is currently approved by the United States Food and Drug Administration for pregnancy prevention for 3 years [2]. However, clinical trials demonstrate high efficacy through 5 years, with no pregnancies reported in the literature during years 4 and 5 [[3], [4], [5]]. This case report describes a pregnancy that occurred during year 4 of implant use in a patient with thyrotoxicosis due to exogenous thyroid hormone replacement.

## 2 Case Presentation

A 41-year-old G2P1 Hispanic patient presented to clinic with an etonogestrel implant in place for four years and five months and an undesired pregnancy. The patient reported monthly menses for the prior 2 years. Her medical history was significant for papillary thyroid carcinoma managed with thyroidectomy and radioactive iodine ablation (RIA) at age 23, repeat RIA at age 26, ulcerative colitis diagnosed at age 25, anxiety, and a term cesarean delivery at age 36. At 1-year post-thyroidectomy, the patient was started on suppressive therapy with levothyroxine 200μg daily because of an elevated level of thyroid stimulating hormone (TSH; 33 mIU/mL); thyroglobulin was normal (19 ng/mL). At 2 years post-thyroidectomy, TSH was <0.03 mIU/mL and free thyroxine was 2.21 ng/dL.

The etonogestrel implant was placed in the postpartum period at age 36. Medications at the time of presentation for undesired pregnancy included levothyroxine 200μg daily, mesalamine 4.8 g daily, and sertraline 50 mg daily. The patient's levothyroxine dose had been unchanged since the implant was placed.

On exam, the patient's body mass index was 23.1 kg/m^2^ and she had had no significant changes in weight over the previous 4 years. An intact implant was easily palpable in the left triceps area. Ultrasonography confirmed an intrauterine gestation of 59 days. On chart review, it was noted that the patient's free thyroxine levels had been frequently supratherapeutic over the previous 4 years (Table 1). The most recent free thyroxine level was measured approximately 3 months before the patient's last menstrual period. The patient reported that her endocrinologist preferred to keep free thyroxine levels above normal as they typically decreased during treatment for ulcerative colitis flares. The patient reported no hyperthyroid symptoms.

**Table 1:** The patient's levels of thyroid stimulating hormone and free thyroxine while she was on thyroid hormone replacement and using the etonogestrel implant.

Thyroid function test	Months after implant placement							
	3	9	21	27	34	36	42	48⁎
Thyroid stimulating hormone (mIU/mL)	<0.02 (L)	<0.02 (L)	<0.02 (L)	<0.01 (L)	0.01 (L)	0.01 (L)	0.01 (L)	0.01 (L)
Free thyroxine (ng/dL)	1.66	1.41	1.17	1.86 (H)	1.83 (H)	2.24 (H)	1.99 (H)	1.96 (H)

H: high; L: low.

Reference ranges: Thyroid stimulating hormone 0.27–4.20 mIU/mL; free thyroxine 0.93–1.70 ng/dL.

⁎ Last results before pregnancy, approximately 3 months before last menstrual period.

The patient chose to have a medication abortion with mifepristone and misoprostol with a one-week follow-up visit for confirmation of outcome and implant removal. At the follow-up visit, transvaginal ultrasonography confirmed gestational sac expulsion. After counseling, the patient opted for implant removal with immediate replacement. Implant removal and replacement after 2–3 years was planned.

## 3 Discussion

There are few case reports of etonogestrel implant failure in years 4 and 5 of use. Etonogestrel used in the contraceptive implant is metabolized during second-pass metabolism by cytochrome *p*450 3A4 hepatic isoenzymes [2]. The biological activity of its metabolites at progestin receptors is unknown [2]. Drug-drug interactions, primarily related to agents that induce cytochrome *p*450 3A4 hepatic isoenzymes, are associated with implant failure [6]. Etonogestrel metabolism is known to be altered by CYP3A4 inducers, including anticonvulsants, barbiturates, rifampin, and St John's Wort, leading to decreased serum concentrations [7]. However, a study of CYP3A4 genetic polymorphisms showed no impact of genetic variations in enzyme function on serum etonogestrel levels [8]. The present patient was not using any medications known to increase etonogestrel metabolism.

Rodents and humans with hypothyroidism have decreased CYP3A4 activity, which affects drug metabolism [8]. Hyperthyroidism has been shown to decrease the half-life of some drugs metabolized by CYP3A4, including digoxin, methimazole, and some beta-blockers [9]; however, a study of 10 patients in an iatrogenic hyperthyroid state found that metabolism of midazolam decreased [10].

In patients with an etonogestrel implant, median serum etonogestrel is 208 pg/mL at three years of use, decreasing to 166 pg/mL at four years of use, and 153 pg/mL at five years of use [5]. One possible explanation for the patient's implant failure is that increased metabolism of etonogestrel due to iatrogenic thyrotoxicosis impacted serum etonogestrel levels in year 4, when levels would have been lower than during the first three years of use, resulting in pregnancy. Although a serum etonogestrel level was not obtained, the implant was kept, and the manufacturer was contacted. However, the manufacturer was not interested in performing residual etonogestrel testing.

In serum, 66% of etonogestrel is bound to albumin and 32% is bound to sex hormone binding globulin (SHBG) [2]. Severe thyrotoxicosis can increase SHBG levels and slightly decrease albumin levels [11,12]. These changes in serum binding proteins may impact implant efficacy, as serum etonogestrel levels decrease with prolonged use. However, given the small effect on binding proteins, we do not suspect that this was the primary mechanism leading to unintended pregnancy in this patient.

There is limited evidence of the effects of hyperthyroidism or excessive thyroid replacement on the metabolism and serum binding of progestins like etonogestrel. It remains possible that this patient simply had a true contraceptive failure or that an unknown risk factor was responsible for her implant failure.

Further study of the impact of hyperthyroid clinical status on progestin metabolism and etonogestrel implant efficacy over multiple years of use would be helpful to fully understand the clinical findings described in this case. This information could aid providers in counseling patients about contraceptive efficacy in unique clinical scenarios.
